# Vitamin B6: A Long Known Compound of Surprising Complexity

**DOI:** 10.3390/molecules14010329

**Published:** 2009-01-12

**Authors:** Sutton Mooney, Jan-Erik Leuendorf, Christopher Hendrickson, Hanjo Hellmann

**Affiliations:** 1 School of Biological Sciences, Washington State University, Pullman, WA, USA; E-mail: suttonmooney@wsu.edu (S. M.), chendrikson@wsu.edu (C. H.); 2 Angewandte Genetik, Freie Universität Berlin, 14195 Berlin, Germany E-mail: j.e.leuendorf@fu-berlin.de (J-E. L.)

**Keywords:** Pyridoxal phosphate, Vitamin B6, Oxidative stress, Derivatives, Transporter.

## Abstract

In recent years vitamin B6 has become a focus of research describing the compound’s critical function in cellular metabolism and stress response. For many years the sole function of vitamin B6 was considered to be that of an enzymatic cofactor. However, recently it became clear that it is also a potent antioxidant that effectively quenches reactive oxygen species and is thus of high importance for cellular well-being. In view of the recent findings, the current review takes a look back and summarizes the discovery of vitamin B6 and the elucidation of its structure and biosynthetic pathways. It provides a detailed overview on vitamin B6 both as a cofactor and a protective compound. Besides these general characteristics of the vitamin, the review also outlines the current literature on vitamin B6 derivatives and elaborates on recent findings that provide new insights into transport and catabolism of the compound and on its impact on human health.

## Introduction

### The Discovery of Vitamin B6

The formula of vitamin B6 (henceforth referred to as vitB6) was first published by Ohdake in 1932. He worked on the isolation from rice-polishings of what he called “Oryzanin” (Vitamin B1) and found vitB6 as a by-product [1,2]. Ohdake described the formula, but he was not aware that he had found a vitamin and did not recognize its physiological importance. 

At this time several scientists worked on the characterization of the vitamin B family members [3,4,5,6,7,8,9,10]. These scientists were searching for the so called “rat pellagra prevention factor” that could cure acrodyna, a pellagra-like skin disorder in rats. They discovered that by adding a special yeast eluate to the diet, acrodyna could be cured. Paul György, a Hungarian born scientist, first described vitB6 as the active “rat pellagra prevention factor” in the yeast eluate [3]. Several years later in 1938 five separate groups of researchers, including György, isolated the crystalline vitB6 from yeast [4,5,6,7,8]. After determination of the structure of vitB6 in 1939, György named the vitamin pyridoxine due to its structural homology to pyridine [9]. In the same year, Stanton A. Harris and Karl Folkers, accomplished the synthesis of vitB6 [10].

**Figure 1:**
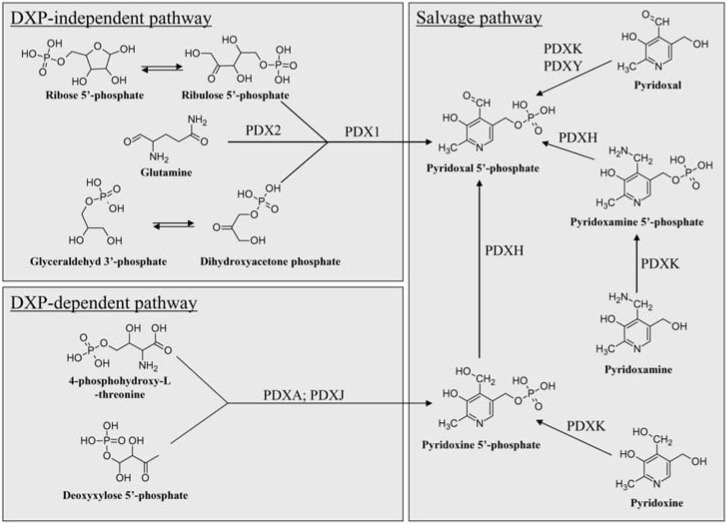
Schematic drawing of *de novo* and *salvage* pathways.

In further studies it was shown that vitB6 could exist in other chemical forms that differ from pyridoxine by a variable group present at the 4′ position [11]. Pyridoxine (PN) carries a hydroxyl, pyridoxal (PL) an aldehyde and pyridoxamine (PM) an amino group (Figure 1). While all three species can be phosphorylated, it is pyridoxal 5’-phosphate (PLP) that is the biologically most active form and used as cofactor for many important enzymatic reactions. 

### The Function of VitB6

The discovery and the first publications on vitB6 ascribed a growth function to the vitamers [3,11]. However, further studies clarified that this relatively rough description understated the diversity of crucial functions and importance for living organism that vitB6 has. VitB6, in the form of PLP, plays a primary role acting as a cofactor for a large number of essential enzymes. These PLP-dependent enzymes catalyze more than 140 distinct enzymatic reactions and belong to five (oxidoreductases EC 1, transferases EC 2, hydrolases EC 3, lyases EC 4, isomerases EC 5) of the six enzyme classes defined by the Enzyme Nomenclature Committee of the International Union of Biochemistry and Molecular Biology (http://www.chem.qmul.ac.uk/iubmb/enzyme). This underlines the wide variety of chemical reactions that PLP-dependent enzymes promote in the organisms and shows again the importance of vitB6. The following section will give an overview of the metabolic reactions in which PLP-dependent enzymes are significantly involved.

Many of the PLP-dependent enzymes catalyze important steps in the amino acid metabolism, like co-catalyzing transamination, racemization, decarboxylation, and α,β-elimination reactions [12,13]. For example, transaminases mediate the conversion of α-ketoacids to amino acids and amino acid racemases produce D-amino acids from L-amino acids [14]. 

Another site of action for the PLP-dependent enzymes is fatty acid metabolism. The enzyme δ-6-desaturase (EC 1.14.19.3) catalyzes the synthesis of vital polyunsaturated fatty acids by the desaturation of linolic acid and γ-linolenic acid, respectively [15,16]. 

Besides these roles, PLP also represents an important cofactor for the degradation of storage carbohydrates, such as glycogen. The PLP-dependent glycogen phosphorylase (EC 2.4.1.1) mediates the glycogen breakdown by the release of glucose from glycogen [17].

Furthermore, two PLP-dependent enzymes are involved in hemoglobin formation and chlorophyll biosynthesis. In these reactions the rate-limiting step is the primary biosynthesis of δ-aminolevulinic acid. In mammals and birds δ-aminolevulinic acid is synthesized by the action of δ-aminolevulinic acid synthase (EC 2.3.1.37) and in plants and algae by the action of glutamate-1-semialdehyde 2,1-aminomutase (EC 5.4.3.8) [18]. 

Additionally, in plants the biosynthesis of the phytohormone ethylene is controlled by the synthesis of the precursor 1-aminocyclopropane-1-carboxylic acid from *S*-adenosylmethionine by PLP-dependent 1-aminocyclopropane-1-carboxylate synthases (EC 4.4.1.14) [19]. 

Apart from its function as a cofactor for PLP-dependent enzymes, vitB6 is also thought to act directly as a protective agent against reactive oxygen species, such as singlet oxygen which will be discussed in a following section [20,21]. 

While fungi, plants, archae, and most eubacteria are able to synthesize vitB6 (see next Section), most animals, including humans, lack this ability and rely on the external supply of vitB6.

## The Known Pathways of VitB6 Anabolism

### Deoxyxylose 5’-phosphate-Dependent and –Independent De Novo Biosynthesis of VitB6

Two existing pathways are known for *de novo* vitB6 biosynthesis. First, the deoxyxylose 5’-phosphate (DXP)-dependent pathway, which is present in eubacteria, such as *Escherichia coli* and second the DXP-independent pathway which is described for some bacteria, archaea and eukarya.

The DXP-dependent pathway has been intensively studied in the gram-negative bacterium *E. coli* [22]. It was shown that in *E. coli,* vitB6 is synthesized by the action of PdxJ (EC 2.6.99.2) and PdxA (EC 1.1.1.262) (Figure 1) [23,24,25]. These two vitB6 synthase proteins use 4-phospohydroxy-L-threonine (4HPT) and DXP, which are precursors in isoprenoid and thiamine biosynthesis, respectively, as substrates to form PNP [26,27,28]. PdxA catalyzes the oxidation of 4HPT to 3-amino-1-hydroxyacetone 1-phosphate (AHAP), and PdxJ forms PNP with the intermediates AHAP and DXP [29]. PNP is then oxidized to PLP, the biocatalytically active form of vitB6, by PdxH via the *salvage* pathway (Figure 1) [30]. The vitB6 precursors 4HPT and DXP originate on the one hand from the oxidation plus transamination of D-erythrose-4-phosphate, and on the other hand, by the synthesis from pyruvate and D-glyceraldehyde-3-phosphate by DXP synthase (EC 2.2.1.7) [31].

Analysis of the crystal structure of the participating enzymes showed that PdxA and PdxJ act separately. PdxA dimers create an interface onto which 4HPT binds [29]. In contrast PdxJ forms octamers as tetramers of PdxJ dimers [32]. In every dimer interface a pocket is located where the intermediates DXP and AHAP are converted to PNP. 

The second known *de novo* vitB6 biosynthesis pathway is the DXP-independent pathway, which is found in bacteria, archaea, and eukarya [13,33,34]. The occurrence of this pathway is demonstrated in plants, fungi, *Plasmodium falciparum*, *Thermotoga maritima* as well as *Bacillus subtilis* and involves two proteins, PDX1 and PDX2 (for pyridoxine biosynthesis protein; orthologs for *B. subtilis* YaaD and YaaE, *Geobacillus stearothermophilus* PDXS and PDXT, *Saccharomyces cerevisiae* SNZ and SNO) [33,34,35,36,37]. These two synthase proteins directly synthesized PLP from ribose 5’-phosphate or ribulose 5’-phosphate, in combination with either glyceraldehyde 3’-phosphate or dihydroxyacetone phosphate and glutamine (Figure 1). Here PDX2 acts as a glutaminase, which deaminates glutamine to glutamate in order to supply nitrogen for the PLP heterocycle, and then PDX1 arranges the final ring closure [33,38].

Crystallization studies in the organisms *B. subtilis, G. stearothermophilus* and *P. falciparum* demonstrated that the PDX enzymes form a synthase complex with a cogwheel-like structure [37,39,40]. The core of the PLP synthase complex consists of 12 PDX1 enzymes that interact in two hexameric layers joining face-to-face to form a dodecamer onto which 12 PDX2 monomers attach [37,39,40]. 

### The Salvage Pathway

In addition to the direct synthesis of new PNP or PLP, the vitamers are interconvertible via the so called *salvage* pathway. These conversions are accomplished by the action of either kinases or oxidases [22,41,42]. The *salvage* pathway has been best analyzed in *E. coli*, where it has been shown that two different kinases (EC 2.7.1.35) can phosphorylate PN, PL and PM to their respective 5’-phosphates (Figure 1). The two kinases differ in their substrate specifities, with PdxY acting on PL, whereas PdxK can utilize all three non-phosphorylated vitamers as substrates. Most eukaryotes contain a single kinase, and crystal structure of the kinase from several different organisms has revealed a dimer [43,44,45,46]. Each of the two monomers contains an active site that utilizes bound ATP and metal ions, which are required for activity [47]. 

Contrary to the kinases, only one oxidase, PdxH (EC 1.4.3.5), is shown in *E. coli* to oxidize the phosphorylated forms of PNP and PMP to PLP (Figure 1) [28,48]. 

In yeast a pyridoxine phosphate oxidase, PDX3 (EC 1.4.3.5), was identified and mutants in this gene had increased oxidative stress sensitivities [49]. Interestingly, complementation with a recently identified oxidase in *Arabidopsis*, AtPPOX, rescued this yeast mutant which underscores the highly conserved nature of the pathway [50]. The crystal structure of pyridoxine oxidase has shown that this protein also functions as a dimer, with the cofactor flavin mononucleotide, FMN, bound in the active site of each monomer [51,52,53].

## The Other End: VitB6 Catabolism

**Figure 2:**
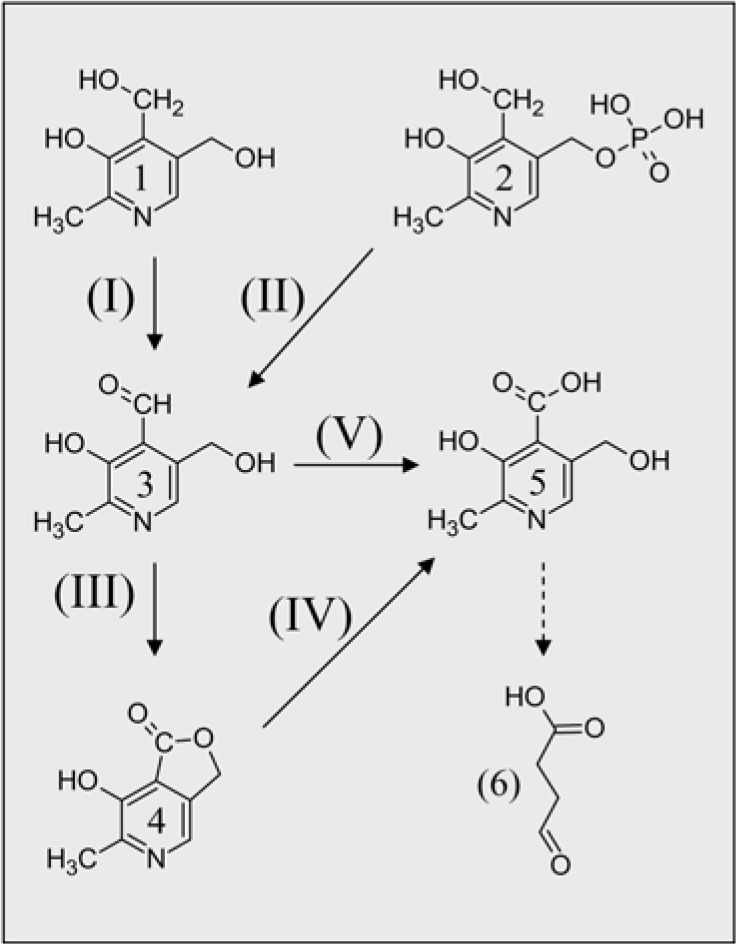
Bacterial catabolic pathways of vitB6. Roman numbers in parenthesis indicate enzymes: (I) pyridoxine 4-oxidase; (II) pyridoxal phosphatase; (III) pyridoxal 4-dehydrogenase; (IV) 4-pyridoxollactonase; (V) pyridoxol oxidase. Arabic numbers indicate compound: (1) PN; (2) PLP; (3) PL; (4) 4-pyridoxolactone; (5) 4-pyridoxic acid; (6) succinic semialdehyde (modified from [62]).

Besides the biosynthesis of vitB6, catabolism of the vitamin is also an important aspect for cellular homeostasis of the compound. A critical step is represented by the dephosphorylation of PLP/PMP/PNP because this step represents a major control for the pool of available active vitB6 cofactor. There are reports of unspecific dephosphorylation of PLP/PMP/PNP by alkaline phosphatase (EC 3.1.3.1) and acid phosphatase (EC 3.1.3.2) [54,55,56,57,58]. However, additional phosphatases have been annotated for various organisms that specifically target phosphorylated vitB6 as a substrate [59,60]. Of these currently the one best characterized is human pyridoxal phosphatase (PLPP) (EC 3.1.3) (Figure 2). The enzyme is a 64 kDa dimer with a requirement for Mg^2+^. It is expressed in various tissues but predominantly in brain, liver and testis [60]. PLPP has its highest affinity for PLP, followed by PNP and then PMP. Inorganic phosphate has a strong inhibitory effect, but the enzyme can also be weakly inhibited by PL [60,61]. Although basic biochemical data are well established for human PLPP control of its activity is still open.

In the gram-negative bacteria *Pseudomonas* sp. and *Mesorhizobium loti* a detailed pathway has been described for degradation of vitB6 and its phosphorylated derivatives (see Figure 2 for an overview) [62,63]. Here PL can either be converted in a two-step process from 4-pyridoxolactone to 4-pyridoxic acid (4-PA) via the subsequent activities of pyridoxal-4-dehydrogenase (EC 1.1.1.107) and 4-pyridoxolactonase (EC 3.1.1.27) or directly to 4-PA by pyridoxal oxidase (EC 1.2.3.8); 4-PA in turn is then catabolized in five additional steps to succinic semialdehyde [62,64]. It is noteworthy that 4-PA is also found in rats and human which makes it likely that similar catabolic pathways exist in these organisms [65,66]. In addition, succinic semialdehyde is a common compound that can accumulate in humans if, for example, degradation of γ-amino-butyric acid is impaired by succinic semialdehyde dehydrogenase (EC 1.2.1.24) deficiency [67]. However, it is basically unknown how the vitamin is degraded in eukaryotes after pyridoxine phosphatase activity.

## Allocation of VitB6 in Prokaryotes and Eukaryotes

A highly important but poorly understood field is the translocation of vitB6 in prokaryotes and eukaryotes. Some vitB6 auxotrophic prokaryotes and single cell eukaryotes rely on the import of the vitamin, while multicellular eukaryotes that cannot synthesize vitB6 need to translocate the vitamin to their different organs. 

Pioneering studies that demonstrated the existence of such vitB6 transporters came from *Salmonella typhimurium* and *S. cerevisiae* [68,69]. In higher eukaryotes studies on rat renal proximal tubular cells demonstrated uptake of pyridoxine and *N*-(4'-pyridoxyl) amines [70]. Here, the work by Zhang and McCormick showed that the tested rat cells have an active Na^+^-dependent uptake system, which is able to discriminate between the different offered substrates. Interestingly, both pyridoxine and its amine derivatives were substrates of a pyridoxal kinase as they were phosphorylated upon entry into the cytosol [70]. Intestinal absorption in humans comes from two sources, dietary ingestion in the small intestine and uptake of bacteria produced vitB6 in the large intestine [71,72]. Experiments using human intestinal epithelial Caco-2 cells and mammalian colonocytes demonstrated the involvement of carrier-mediated systems for both with pH, temperature and pyridoxine levels affecting the rate of transport. An intercellular protein kinase A (PKA)-mediated pathway appears to regulate transport in the Caco-2 cells, whereas transport in colonocytes appears to be regulated by a Ca^2+^/CaM- mediated pathway [71,72]. Work in human placental tissue has also indicated the presence of a passive transport system that transfers pyridoxine in and out of the cells but not against a concentration gradient [73]. Both active uptake and export of vitB6 have been demonstrated in yeast [69,74,75]. The fission yeast, *Schizosaccharomyces pombe,* showed a preferential efflux of PN that was dependent on the internal concentration of PN, and the rate was increased with the addition of Na^+^ [74]. It was suggested that the membrane potential could be affecting the PN efflux gate or carrier. Additionally, work in *S. pombe* has linked the PL reductase encoded by *prl1+* gene to the excretion of PN following its reduction from PL as part of the maintenance of vitB6 levels in the cell [76]. Overall these findings demonstrated the presence of various mechanisms for transport of the vitB6. However the precise identity of the transport proteins that facilitate such movements has remained open. Described for *S. cerevisiae* in 2003, Tpn1p was the first example of a eukaryotic vitB6 transporter [77]. The protein belongs to the family of purine-cytosine permeases and functions as a plasma membrane bound proton symporter for vitB6 uptake. It has a high affinity for PN with a K_m_ value of 0.55 μM but also transports PM and PL with lower affinities. A second unrelated transporter, Bsu1p was identified in *S. pombe,* a yeast strain that does not contain a Tpn1p homlog [78]. While Bsu1p has lower affinity for PN than Tpn1p, it also operates as a proton symporter with similar optimal pH and substrate profiles. Likewise, expression of both transporters increased when PN concentrations decreased [77,78]. Very recently, a novel class of vitamin transporters were identified in prokaryotes [79]. They are composed of different modules that have substrate specific components and an energy-coupling module and were named accordingly as energy-coupling factor (ECF) transporters. The energy-coupling module allows docking of different substrate specific components to facilitate selected transport of metabolites across membranes. Interestingly, the authors also identified an ECF transporter with a high affinity for pyridoxine [79]. The findings of Tpn1p, Bsu1p and the ECF transporters demonstrate that different transport pathways have evolved and it will be exciting to learn what kind of transport proteins are active in other organisms. In addition it will be of importance to understand intracellular transport of vitB6 since many PLP-dependent enzymes are present in mitochondria and chloroplast. However, currently it is open how this is achieved in the cell since corresponding transport proteins remain to be identified.

## The Role of VitB6 in Stress Response

Recent work has provided evidence of an expanded role of vitB6 in cells. As stated above, this compound serves a role in enzymatic catalysis. However, vitB6 may play a crucial role in protecting cells from oxidative stress because the vitamin has been shown to exhibit antioxidant activity that even exceeds that of vitamins C and E [80,81,82,83]. Upon mutation of genes involved in the *salvage* and *de novo* pathways of vitB6 synthesis, a range of phenotypes are seen in salt and reactive oxygen species (ROS) sensitivity (Table 1). ROS sensitivity in context with vitB6 was originally characterized in the phytopathogen *Cercospora nicotianae*. Here mutant strains were identified that were particularly vulnerable to their own toxin cercosporin, a photosensitizer that produces singlet oxygen and superoxide upon irradiation [35,81]. Cloning of the mutant genes in *C. nicotianae* revealed that the mutated fungi were affected in a PDX1 ortholog [35,80,81]. These findings were of importance since so far vitB6 had not been mentioned in the context of singlet oxygen resistance [84]. Subsequent studies in other organisms also showed that vitB6 is crucial for oxidative stress tolerance and other abiotic stressors. For example, loss of *Arabidopsis thaliana* PDX1.3 causes hypersensitivity towards treatment with Rose Bengal, a ROS inducing chemical [85]. Moreover *Arabidopsis pdx1.3* mutants are also hypersensitive towards salt and UV-B treatments [85,86]. It is also interesting to note that mutants in the *salvage* pathway show aberrant stress sensitivities, demonstrating that vitB6 metabolism in general is critical for abiotic stress tolerance. For example, the *Arabidopsis sos4* mutant that is affected in pyridoxine kinase PDXK is highly sensitive to salt stress [87]. However, unlike mutants affected in *PDX1* genes that have been demonstrated to contain reduced levels of vitB6, *sos4* has overall increased levels of the vitamin [86,88,89,90]. Though the precise reason for increased vitB6 levels in *sos4* remains to be answered, the example given demonstrates that vitB6 levels do not strictly correlate with stress tolerance. Hence, one must question whether aberrant vitB6 levels are the primary reason for the observed abiotic stress sensitivities.

It is noteworthy that *PDX1* and *PDXK* gene expression is also regulated in response to abiotic stress. For example, *B. subtilis PDX1* has been demonstrated to be upregulated in the presence of the singlet oxygen producer, methylene blue [91]. In addition, *PYRO A* (a *PDX1* homologue described in *Aspergillus nidulans)* and *Arabidopsis* PDX1.3 are upregulated after exposure to UV radiation [92,93]. Also SNZ1, a *S. cerevisiae* PDX1 homologue, has been shown to be present in higher amounts during the stationary growth phase in which cultures are more prone to oxidative stress [94,95]. Findings in plants revealed that expression of *Arabidopsis PDX1* genes is regulated by drought, chilling, UV-B treatment, and ozone [96]. Finally, *SOS4* up-regulation has also been demonstrated as a response to cold stress and abscisic acid (ABA) treatment [87]. 

Overall there appears to be a broad and beneficial effect of vitB6 on abiotic stress tolerance in the cell, and stressors have been found to result in an increased amount of expression of genes involved in vitB6 biosynthesis. Notably and as mentioned above there are some exceptions to the observation that increased vitB6 availability is beneficial: Herrero and Daub observed negligible changes in vitB6 content in tobacco in response to salt stress, and Gonzalez and coworkers noted significantly higher vitB6 levels in *sos4* relative to wild type plants despite the mutant’s increased salt sensitivity [90]. Considering these findings, future work may aim to link additional signals to a response of altered vitB6 production in cells. Additionally, characterization of the ROS quenching capacity and regulation of vitB6 biosynthesis may help to solve the close relationships seen between the vitamin and the described sources of stress. 

**Table 1:** Examples of VitB6 *De Novo* and *Salvage* Pathway Mutants in Context with Stress.

Organism	Mutant	Pathway affected	Phenotype	Citation
*E. coli*	*ppox/pdxH*	*Salvage*	Reduced growth, aberrant shape	[27]
*C. nicotianae*	*sor1/pdx1*	*De novo*	Increased ROS sensitivity, loss of vitB6 production, increased salt sensitivity, reduced growth	[81]
*S. cerevisiae*	*snz1/pdx1*	*De novo*	Reduced growth in minimal media	[94]
*S. cerevisiae*	*sno1/pdx2*	*De novo*	Reduced growth in minimal media	[94]
*S. cerevisiae*	*pdx3*	*Salvage*	Increased ROS sensitivity	[50]
*A. thaliana*	*sos4-1*	*Salvage*	Increased salt sensitivity	[87, 90]
*A. thaliana*	*pdx 1.1, pdx 1.3*	*De novo*	Increased salt sensitivity	[21, 86]
*A. thaliana*	*pdx3/PPOX*	*Salvage*	Reduced aerial & root growth, increased salt sensitivity	[50, 90]

## The Diversity of VitB6 Derivatives

As described in the preceding paragraphs, vitB6 is a well-investigated compound critical for many cellular processes as either a central cofactor or as a potent antioxidant. However, it is noteworthy that a variety of different PN, PM, and PL derivatives have been described, for which the precise function is not understood (Table 2). These derivatives potentially have novel functions, and may be crucial to fully appreciate the biological relevance of vitB6.

The best known of these derivatives is probably 4’-*O*-methylpyridoxine or ginkgotoxin from the tree *Ginkgo biloba* [12,97]. The compound has been found in different tissues with the highest concentrations being present in seeds [98]. Although it has been shown that the additional 4'-*O*-methyl group most likely derives from methionine, and that both phosphorylated and non-phosphorylated forms of pyridoxine are methylated, the biosynthetic pathway leading to 4’-*O*-methylpyridoxine is still unresolved [98,99]. Ingestion of the toxin can lead to *Gin-nan-sitotoxism*, epileptic convulsions, and other neuronal disorders [100]. As seeds from *Ginkgo* trees are a food source in China and Japan, and extracts from leaves are used in pharmaceutical products, they represent a potential health risk. The PLP-dependent enzyme glutamate decarboxylase (GAD), which is critical for synthesis of the neurotransmitter GABA was discussed as a potential target of 4’-*O*-methylpyridoxine. However, there is no clear evidence that ginkgotoxin significantly reduces GAD activity when present in physiologically relevant concentrations [101]. In contrast, recent work rather suggests that the toxin is competing with PN/PM/PL for human pyridoxine kinase [97]. This in turn might reduce the pool of available PLP and PMP in the brain and negatively affect GAD activity and GABA biosynthesis [97]. 

Ginkgotoxin was also found in the African tree *Albizia tanganyicensis* [98,99,100] demonstrating that the biosynthetic pathway leading to the formation of 4’-*O*-methylpyridoxine is not unique to *Ginkgo*. *Albizia tanganyicensis* and its close relative *Albizia julibrissin* also synthesize other more complex vitB6 derivatives (see Table 2) [100,102]. Unfortunately, neither for ginkgotoxin nor for the other *Albizia* derivatives could we find a biological function explaining why these compounds are synthesized. A likely possibility is that they serve as protecting compounds against pathogens due to their toxicity. This poses the attractive question as to what kind of mechanisms these plants employ to protect their own metabolism against toxic vitB6 derivatives. For example, do they utilize specific compartments or organelles for storage of their toxic compounds?

Another aspect that vitB6 derivatives have been brought in context with is the formation of advanced glycation and lipoxygenation end-products (AGE and ALE, respectively). AGE and ALE formation can occur in cells when reduced sugars (e.g. glucose, fructose) or polyunsaturated fatty acids are abundant. In such a situation they can cross-react preferentially with lysine residues of proteins [103,104]. Accumulation of AGE and ALE is also caused by oxidative stress or overload of pathways active in detoxification [103]. Such end-products are often detrimental for protein function and, especially in older tissues, might lead to severe damage. Hence, patients with diabetes or atherosclerosis that have increased contents of blood sugar or blood lipids, respectively, suffer from accumulation of AGEs and ALEs. Here, pyridoxamine is discussed to serve as a protecting compound by bonding with fatty acids (see Table 2) and thereby effectively competing with proteins for ALE formation [104,105]. The vitamin is also discussed to serve as a protecting compound for AGE formation which is of special interest to patients suffering from diabetes [106,107,108]. 

A significant proportion of vitB6 (ranging from 5-80% of the total vitB6 content) in many fruits and vegetables is glycosylated [109,110]. Glycosylated vitB6 appears to be abundant in plants and has been detected in soybean, rice, and *Ginkgo* [111,112,113]. Furthermore, in fungi β-fructosyl and β-galactosyl compounds of pyridoxine have been found [114,115]. It is likely that these derivatives of vitB6 are not substrates of a pyridoxine kinase and thus are not accessible for metabolic utilization. Consequently, specific β-glucosidases have been described in plants and human capable of removing the sugar moiety, making the vitamin again accessible for *salvage* pathway enzymes [111,116,117,118]. Although no precise explanation for the high amount of glycosylated vitB6 is provided in literature, a possibility can be seen in context with AGE accumulation. Here, vitB6 might serve as a protecting compound to prevent reaction of sugar with lysine residues of proteins. Alternatively, glycosylated forms of vitB6 might serve as storage compounds of the vitamin and even carbohydrates that can be mobilized upon demand. Overall the existence of such a diverse variety of vitB6 derivatives indicates that the vitamin is involved in or employed for many other currently unknown processes. 

**Table 2:** Examples of VitB6 Vitamers and Their Derivatives

Derivative	Structure	Function	Organism found	citation
Vitamin B6	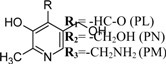	Antioxidant	ubiquitous	[13]
Vitamin B6-phosphate	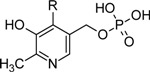	Cofactor	ubiquitous	[13]
4’-*O*-Methyl-pyridoxine (ginkgotoxin)	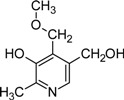	Unknown; potential inhibitor of PN/PM/PL kinase	*Ginkgo biloba*, *Albizia tanganyicensis*	[23, 97, 99]
5’-*O*-Acetyl-4’-*O*-methylpyridoxine	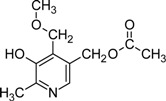	Unknown	*Albizia tanganyicensis*	[100]
Julibrine I	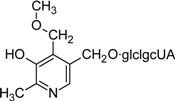	Unknown	*Albizia julibrissin*	[102]
Julibrine II	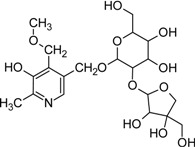	Unknown; has been demonstrated to induce arrhythmia	*Albizia julibrissin*	[102]
5'-0-(β-D-Glucopyranosyl) pyridoxine	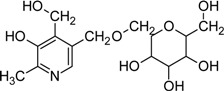	Unknown	Various plant products	[111, 112]
*N*-Hexanoyl-pyridoxamine (HAPM)	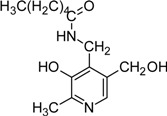	Inhibitor of advanced glycation and lipoxidation reactions	PM treated diabetic and obese rats	[9, 10]
*N*-Nonanedioyl-pyridoxamine monoamide (NDAPM)	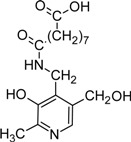	Inhibitor of advanced glycation and lipoxidation reactions	PM treated diabetic and obese rats	[9, 10]
*N*-Pentanedioyl-pyridoxamine monoamide (PDAPM)	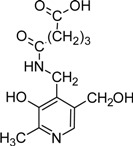	Inhibitor of advanced glycation and lipoxidation reactions	PM treated diabetic and obese rats	[104, 105]
N-Formyl-pyridoxamine (FAPM)	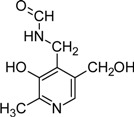	Inhibitor of advanced glycation and lipoxidation reactions	PM treated diabetic and obese rats	[104, 105]

## VitB6 Supplements and Human Health: Is it too much or not enough of a good thing?

The importance of vitB6 in human health is reflected in how actively it is studied in relationship to a wide range of disease prevention and control. The current *Recommended Dietary Allowance* (RDS) from the American National Institute of Health (NIH) for vitB6 is 2 milligrams per day with an upward tolerance of 100 mg per day for adults. High doses can lead to peripheral sensory neuropathy and nerve degeneration [119,120]. These problems are generally reversible when supplementation is stopped. Additionally some studies have suggested that increased levels of the B6 vitamers and some derivatives can generate toxic photoproducts as a result of UV irradiation [121,122,123]. 

Most problems are stemming from deficiencies of the vitamin and there are several areas of interest actively being pursued. Numerous clinical trials have been conducted to observe the broader impact of vitB6 on human health problems such as cancer prevention and recovery and the amelioration of neurological diseases. Also under investigation are the benefits of increased vitB6 through its role as a cofactor in upstream processes that lead to problems involving heart disease, osteoporosis and diabetes. Several of these topics are addressed in the following section. 

Studies of vitB6 supplements in cancer prevention have yielded mixed results ranging from no significant benefits seen in breast cancer to indications of protection against colorectal cancer [124]. A recent study on hairless mice given PN supplements was performed to see if increased vitB6 protected against UV-B induced skin tumors [121]. It was reasoned that vitB6 would help quench reactive oxygen species (ROS), which are associated with the development of cancer. Interestingly though, while higher serum levels of PLP correlated with a higher dosage of dietary PN, neither the amount of PLP nor oxidative stress markers in skin differed in relationship to the dosages. Additionally an increase in tumor induction was seen in the mice given the higher doses of PN, correlating with previous work [122,123]. Interestingly decreased levels of vitB6 accompanied by increased levels of oxidative stress were detected in red blood cells of non-small cell lung cancer patients, also highlighting the vitamin’s potential benefits as an antioxidant [125].

Phosphorylated vitB6 is needed as a cofactor for neurotransmitter synthesis. However, studies on elderly people have shown that a walking program has more benefits on cognitive improvement and increased folate may help prevent Alzheimer’s diseases rather than vitB6 supplements [126,127,128,129]. While an association of low PLP and high symptoms of depression have been reported, supplementation with vitB6 has not been shown to conclusively improve depression in older men [130,131]. In schizophrenic patients with tardive dyskinsia, plasma levels of PLP were significantly lower and treatment with vitB6 supplements reduced the symptoms of this disease along with another schizophrenic associated disease, akathisia [132,133,134]. VitB6 is also studied as a potentially important candidate to improve behavioural disorders of autistic children, although the precise impact of the vitamin remains to be shown. [135,136]. 

VitB6 is involved in maintenance of normal homocysteine levels, and lower levels of homocysteine are associated with lower rates of coronary heart disease and stroke [137]. However studies are conflicting as to whether giving supplements to lower the homocysteine levels improves protection against these diseases [137,138,139]. High homocysteine levels have also been linked to osteoperosis and bone fragility fractures. *In vitro* tissue experiments demonstrated that either decreasing vitB6 or increasing homocysteine levels stimulated osteoclast activity, which leads to bone resorption [140]. 

Blood vitB6 levels are significantly decreased in diabetics. As vitB6 is a cofactor in tryptophan catabolism, disruption of this pathway leads to increased levels of kynurenine metabolites which inhibit insulin secretion and lower glucose tolerance [141]. Studies on different B6 vitamers have shown that supplements can help with problems related to glucose tolerance [142]. Complications from other diabetes-associated diseases are also common, and interestingly high doses of vitB6 normalized endothelial dysfunction, a precursor to vascular disease, in children with type 1 diabetes [143].

## Outlook and Perspectives

Though in the last years various aspects in vitB6 biosynthesis and the impact and benefits of the vitamin for general metabolism have been explained, many questions remain unanswered. Five of these are covered in the following section to provide an outlook on future opportunities related to this important research field.

*Do regulatory mechanisms exist that control PLP de novo biosynthesis?* Although the two biosynthetic pathways – DXP-dependent and –independent – have been resolved, regulatory mechanisms on the transcriptional and posttranscriptional level to control PLP biosynthesis remain to be found. Although transcriptional regulation of *PDX1* genes has been described for various organisms after stress treatments, it remains open whether this in turn affects vitB6 levels [13,144]. Considering the central and indispensable role of PLP in metabolism one would expect that such regulatory switches exist. These need to be connected with general metabolism, first because vitB6 exhibits such a central role as a cofactor and second because the *de novo* biosynthetic machinery competes for the precursors required for PLP synthesis with other pathways. 

*How are the salvage pathway and PLP phosphatase activities regulated?* As for the *de novo* pathways, it has not been explained how *salvage* pathway enzymes and PLP phosphatases are regulated. This is surprising as pyridoxine kinase and PNP/PMP oxidase are crucial players in controlling vitB6 homeostasis and the availability of the active cofactor which might even occur in concert with PLP phosphatases. However, only a few studies reveal insights on factors like ions, ATP, or end-products that directly affect activities of these enzymes [61,145,146,147,148,149], while the interplay between the different proteins and the *de novo* pathway has not been addressed. Again, one would suggest that some higher degree of active control is present that regulates the proteins of the *salvage* pathway and PLP phosphatases upon demand. 

*What are the mechanisms of vitamin B6 translocation*? For many organisms it is open how vitB6 is translocated within the different organs and tissues. Although the non-phosphorylated forms can to some extent go passively through membranes, this diffusion is most likely insufficient for long distance allocation or rapid uptake of the vitamin when needed [77]. Currently only a few examples are given for vitB6 transporters in yeast and prokaryotes, making this an important question in other organisms like animals or plants [77,79].

*How does vitamin B6 metabolism positively affect stress tolerance?* For many organisms it has been shown that mutants affected in either the *salvage* or the *de novo* pathway are hypersensitive towards abiotic stress conditions. However, this hypersensitivity does not always correlate with the vitB6 content in the cell [86,90]. Hence it is currently unclear and necessary to ask whether the amount of vitB6 is the critical factor to protect against abiotic stress, whether it is the vitB6 homeostasis that is important, or whether it is the proteins that participate in the different pathways that have additional functions connected to stress alleviation.

*Are there more de novo pathways present in yet unexplored organisms and how are vitB6 derivatives formed?* Currently it appears to be that there are just the two described *de novo* pathways, and no evidence for additional biosynthetic pathways is at hand. Though one cannot exclude the possibility for a third pathway, it is more likely that only the two described DXP-dependent and DXP-independent pathways exist. In contrast the variety of existing vitB6 derivatives indicates an extensive metabolic ability of organisms to modify vitB6. It will be interesting in the future to have more knowledge generated on the enzymes that modify vitB6, about the biological purposes of these compounds, and how these organisms protect themselves against potentially toxic derivatives. Understanding these points may provide better approaches to utilizing the pharmaceutical potentials of vitB6 and its derivatives for human health. 
